# Prevalence of malaria and clinical profile of febrile HIV infected patients in three HIV clinics in Ivory Coast

**Published:** 2017-11-01

**Authors:** Yapo T. Aba, Raoul Moh, Nogbou F. Ello, Serge-Brice Assi, Ama M. Ano, Brigitte Koffi, Mélaine C. Mossou, Zelica Diallo, Emmanuel Bissagnene

**Affiliations:** 1 Department of Infectious and Tropical Diseases, University of Alassane Ouattara, Bouaké, Ivory Coast; 2 Department of Infectious and Tropical Diseases, University of Felix Houphouet Boigny, Abidjan, Ivory Coast; 3 Pierre Richet Institute, Bouaké, Ivory Coast.; 4 National Institute of Public Health, Abidjan, Ivory Coast

## Abstract

**Background:**

To determine the prevalence and clinical profile of malaria among febrile HIV-infected patients followed up in three HIV clinics in Ivory Coast.

**Materials and methods:**

A cross-sectional multicentre study was conducted between 2009 and 2010 in the Pneumology Department of Cocody Teaching Hospital in Abidjan, Medical Esperance Centre and the Regional Hospital in San-Pedro. Patients of all ages presenting with fever (rectal or axillary temperature >37,5°C) or a medical history of fever within 72 hrs prior to consultation were included. Parasitological diagnostic methods used were microscopy by blood smear (BS) for search malaria parasite and parasite density. Haemoglobin levels were assessed to assess anaemia.

**Results:**

Over the study period, 530 people living with HIV consulted for fever. The 476 patients included were predominantly female (n=280, 59%), with a median age of 34 (range 3-74 yrs), a mean of 38 ± 8.3 (SD) yrs, infected with HIV-1 (n=409, 86%), on antiretroviral therapy (n=376, 79%), and cotrimoxazole prophylaxis (n=381, 80%). Only 73 (15%) patients were using LLINs. Malaria prevalence was 10% (n=47). *Plasmodium falciparum* was the only species identified with a mean density of 15 900 trophozoites/μl. Malaria was more common among patients with a CD4 count of <200/mm^3^ (p<0.001) neither on cotrimoxazole prophylaxis (p<0.001) nor on antiretroviral therapy (ART) (p<0.001). Uncomplicated malaria accounted for 32 (68%) of the cases. The signs of severe malaria (n=15, 32%,) were dominated by severe anaemia (n= 12, 25.5%).

**Conclusion:**

Our study revealed that malaria prevalence appears to be low in HIV clinics for people living with HIV on HAART and cotrimoxazole prophylaxis. Uncomplicated malaria is predominant when consultation is early. Signs of severe malaria were dominated by severe anaemia.

## 1 Introduction

Malaria and HIV infection are rampant, mainly in sub-Saharan Africa, where both diseases constitute major public health problems [1]. In Africa there is geographical, epidemiological and pathogenic overlap between *Plasmodium* and HIV, resulting in co-infected patients [2-4]. Yet their comorbidities vary according to prevalence and mortality [5 -7]. Côte d’Ivoire is one of the sub-Saharan countries affected by the HIV pandemic, with a prevalence of 3.7% [8]. In this country, until 2011, malaria diagnosis was mainly based on clinical presumption in health facilities [9]. This contributed to overestimation of malaria cases, and consequently overconsumption of antimalarial drugs. Indeed, studies have addressed the HIV/malaria co-infection in clinical trials assessing the protective effect of cotrimoxazole [7], but the actual prevalence and clinical presentation of malaria are poorly studied in the population of HIV-infected patients in Côte d’Ivoire. This in spite of the fact that malaria is quoted as the third cause of HIV-related morbidity in Africa [10]. The purpose of our study was therefore to determine the prevalence and clinical profile of malaria among febrile HIV-infected patients in HIV clinics, using the then in the country available diagnostic methods (microscopy and blood smears) prior to the now common general use of rapid diagnostic tests (RDTs).

## 2 Materials and methods

### 2.1 Study area

Côte d’Ivoire is a malaria-endemic country of stable transmission with an upsurge of cases during the long and short rainy seasons between April to mid-July and mid-July to September, respectively [11]. Our study was conducted simultaneously in three HIV-clinics for people living with HIV (PLHIV) from two cities where the prevalence of malaria and HIV infection was high [12,13]: Abidjan, located in south of Côte d’Ivoire, lagoon area and San Pedro located in forest area in the southwest, 348 km from Abidjan. The HIV -clinics were the HIV–clinic of Pneumo-phthisiology (PPH) department of Cocody Teaching Hospital in Abidjan, Medical Esperance Centre (MEC) and the Regional Hospital (RH) of San Pedro (the two HIV-clinics of San-Pédro).

At the time of the survey, among the 4661 PLHIV in the database of the PPH department of Cocody Teaching Hospital, 2249 patients were on antiretroviral therapy (ART) and the active file (defined as the number of patients being followed up) was 825 patients. The MEC in San Pedro had 1578 patients in its database of whom 800 were on ART, in an active file of 674 patients. In the RH of San Pedro, of the 2201 patients enrolled in the database, 1163 patients were on ART, in an active file of 1063 patients. In 2009, prior to our study, the annual report on malaria from HIV clinics indicated 341 cases in department of PPH and 667 cases in San-Pedro.

### 2.2 Study type, patients and diagnosis

A cross-sectional multicentre study was carried between December 2009 and September 2010. The target population consisted of febrile HIV-patients from the active files of the three HIV clinics mentioned above. Patients of all ages presenting with fever (rectal or axillary temperature >37.5°C) or a medical history of fever within 72 hrs prior to consultation were included. Conversely, patients who were taking or had taken antimalarial drugs within 14 days preceding the inclusion day were excluded because prior studies have shown an efficacy of antimalarials of 97-99% during 28-day follow-up in Ivory Coast [14,15].

Six months prior to the study, awareness/information sessions about the study were conducted during routine quarterly visits of patients from the active files of the three HIV-clinics, inviting them to go to their clinic in case of fever. Social, clinical and HIV data were collected. Long-lasting insecticide-treated bednet (LLIN) usage was assessed. Microscopy of blood smears (BS) for parasite identification and density were performed in accordance with the national guidelines of malaria diagnosis [16]. CD4 counts were monitored at the time of inclusion to update the routine monitoring report. Haematological and biochemical tests (blood glucose, creatinine, transaminases) and microscopy for BS were performed in the hospital laboratory. Uncomplicated malaria and severe malaria were defined by the presence of clinical signs and asexual forms of *P. falciparum* in the peripheral blood smear according to WHO classification [17]. The value of haemoglobin was assessed with the haemogram test to determine anaemia.

Thick smears were Giemsa® stained in the field and examined under an oil immersion using a microscope (ocular 10X, lens 100X). BS were fixed in pure methanol before staining. *Plasmodium* species were identified and asexual forms of each species counted on 200 leucocytes. Parasite density was calculated by assuming an average of 8,000 leucocytes/μl of blood.

### 2.3 Data analysis

Data were collected using a standard survey form and were analysed using Epi Info 3.5.1 software. The distribution of quantitative variables was described by the mean, standard deviation and interquartile range. Conversely, qualitative variables were described as total number and percentage. The Chi-square and Fisher’s exact tests, when appropriate, were used to compare proportions (with Yates correction). All tests were performed with a 5% significance threshold. The risk factors associated with malaria occurrence, severity and mortality were investigated using univariate analysis.

### 2.4 Legal and ethical aspects

This study was implemented in accordance with the national guidelines of the National Malaria Control Programme (NMCP) [16]. No HIV testing was performed. Patients were already known to be infected with HIV and followed in the three study clinics. The CD4 values obtained served to update the routine monitoring report. Data confidentiality was guaranteed by assigning anonymous study numbers to patients in ascending and chronological order of inclusion.

## 3 Results

**Figure 1:**
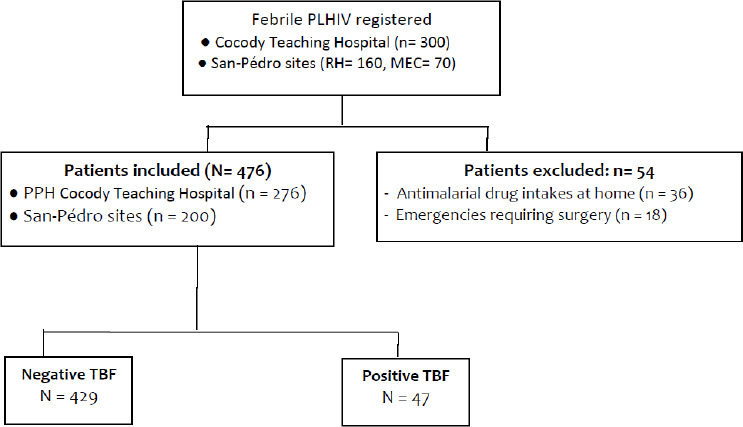
Study patient flow diagram (PLHIV: People Living with HIV, MEC: Medical Esperance Centre, PPH: Pneumo-phthisiology, RH: Regional Hospital, TBF: thick blood film).

Over the study period, 530 PLHIV consulted for fever, which included 300 patients from the active file of the PPH department of Cocody Teaching Hospital and 230 patients from both San Pedro sites. Of these patients, 54 were not included in the study because they administered antimalarial treatment at home or were emergencies requiring surgery (Figure 1)**.** The 476 patients included were predominantly female (59%). Their ages ranged between 3 and 74 yrs with a median of 34 yrs and mean of 38 ± 8.3 (SD) yrs. Five percent of the patients (n= 24) were below 15 yrs of age.

A minority of patients (n=71, 15%) used an LLIN at home for malaria prevention (Table 1)***,*** and LLINs were less frequently used in Abidjan than in San Pedro (2% vs 34%, p <0.001).

**Table 1:** Patient profile according to the study site.

Parameters	PPH Cocody teaching hospital site n=276	San-Pedro sites n=200	Total n= 476
Mean age (yrs) ± SD*	40 ± 10.2	36 ±11.3	38±8.3
Female	146 (53%)	134 (67%)	280 (59%)
Primary education level	168 (61%)	54 (27%)	222 (47%)
LLIN use	5 (2%)	68 (34%)	73 (15%)
HIV-1 Infection	225 (81.5%)	184 (92%)	409 (86%)
CD4 count < 200/mm^3^	77 (28%)	72 (36%)	149 (31%)
Patients on cotrimoxazole	259 (94%)	122 (61%)	381 (80%)
Antiretroviral therapy	220 (79.7%)	156 (78%)	376 (79%)
- 2 NRTI + 1 NNRTI**	167 (60.5%)	139 (69.5%)	306 (64.3%)
- 2 NRTI + 1 PI/r	33 (12%)	16 (8%)	49 (10.3%)
- 3 NRTI	20 (7.2%)	1 (0.5%)	21 (4.4%)
Comorbidities			
- S+PTB patients on treatment	8 (3%)	0 (0%)	8 (1.7%)
- High blood pressure	3 (1%)	5 (2.5%)	8 (1.7%)
- Diabetes	3 (1%)	1 (0.5%)	4 (0.8%)
- Cerebral toxoplasmosis patients on treatment	3 (1%)	0 (0%)	3 (0.6%)
- Hb SS Sickle cell disease	1 (0.4%)	0 (0%)	1 (0.2%)
- CMV retinitis patients not on treatment	1 (0.4%)	0 (0%)	1 (0.2%)
- Malignant non-Hodgkin’s lymphoma	1 (0.4%)	0 (0%)	1 (0.2%)
- Asthma	0 (0%)	1 (0.5%)	1 (0.2%)

* Standard deviation, ** NNRTI: Non-nucleoside reverse transcriptase inhibitor, NRTI: Nucleoside reverse transcriptase inhibitor, PI: Protease inhibitor, S+PTB: Smear-positive pulmonary tuberculosis, CMV: Cytomegalovirus, PPH: Pneumo-phthisiology, Hb: Haemoglobin.

Twenty-five patients (19 from the PPH site and 6 from the San Pedro sites) presented underlying diseases (5%) with a predominance of smear positive pulmonary tuberculosis patients on treatment (S+PTB, n=8) and high blood pressure (n=8). HIV-1 infection was predominant at 86% versus 1% for HIV-2 and 13% for HIV-1+2. The median duration of patient care in HIV clinics was 14 months [range 1– 49 months]. At the time of inclusion in the study, 31% were very immunosuppressed with a CD4 count < 200/mm^3^; 79% were on first or second-line antiretroviral therapy including 10.3% on protease inhibitors and 80% (n=381) on cotrimoxazole prophylaxis. The median duration of cotrimoxazole prophylaxis was eight months [range 7 days - 42 months]. That of the antiretroviral treatment was eight months [range 10 days - 43 months]. About 81% of ongoing treatment regimens contained zidovudine (Table 1)**.**

Of the 476 patients included, the positivity rate of TBF was 10% (n=47). Only *P. falciparum* was identified, with a mean parasite density of 15 900 trophozoites/μl of blood (range 98 - 82 000 trophozoites/μl). Body temperature ranged from 37.8°C to 43°C with an average of 38.7° C.

Uncomplicated malaria accounted for 32 (68%) cases and severe malaria for 15 (32%), with a predominance of severe anaemia defined by haemoglobin level < 5 g/dl (n=12 cases). Convulsions (>2/24 hrs, 5 cases), creatinine >265 mmol/l (5 cases), Glascow coma scale <9 (4 cases) and Jaundice (2 cases) were observed. Among severe malaria cases, other diseases were reported such as pneumococcal meningitis (1), sickle cell crisis (1) and smear-positive pulmonary tuberculosis (3). Likewise, active intercostal zoster (1), unbalanced diabetes (2), cytomegalovirus retinitis (1) and cerebral toxoplasmosis (3) were reported among patients with uncomplicated malaria. Malaria was more frequently observed in severely immunosuppressed patients with a CD4 count < 200 (p<0.001), patients not on cotrimoxazole prophylaxis (p<0.001) and patients not on ART (p<0.001) (Table 2). Conversely, there was no significant association between severe malaria and any of the following factors: CD4 count < 200/mm^3^, absence of cotrimoxazole chemoprophylaxis and absence of ART (Table 3).

**Table 2:** Factors associated with the occurrence of malaria.

Parameters	Malaria present n=47	Malaria absent n=429	OR	95% CI	p
CD4			3.44	1.78-6.65	< 0.001
< 200	27 (57%)	121 (28%)			
≥ 200	20 (43%)	308 (72%)			
Cotrimoxazole			47.45	18.19-131.03	< 0.001
Not prescribed	41 (87%)	54 (13%)			
Prescribed	6 (13%)	375 (87%)			
ART*			5.94	3.04-11.65	< 0.001
Not prescribed	26 (55%)	74 (17%)			
Prescribed	21 (45%)	355 (83%)			
LLIN use			0.63	0.24-1.65	0.466
Yes	5 (11%)	68 (16%)			
No	42 (89%)	361 (84%)			

* Antiretroviral therapy

**Table 3:** Factors associated with the occurrence of severe malaria.

Parameters	Severe malaria present n=15	Uncomplicated malaria n=32	OR	95% CI	p
CD4			1.76	0.42-7.71	0.38
< 200	10 (67%)	17 (53%)			
≥ 200	5 (63%)	15 (47%)			
Cotrimoxazole			0.41	0.05-3.09	0.58
Not prescribed	12 (80%)	29 (91%)			
Prescribed	3 (20%)	3 (9%)			
ART*			2.50	0.60-10.66	0.15
Not prescribed	9 (60%)	12 (38%)			
Prescribed	6 (40%)	20 (62%)			

* Antiretroviral therapy

## 4 Discussion

Developed to cover one full year, the study could only be carried out over a period of ten-month (December 2009-September 2010) because of the military-political turmoil experienced in Côte d’Ivoire during the presidential election period around October 2010, which poses a limitation to the study. However, it has the merit of including the two rainy seasons, in which we observed an increase in malaria cases [11]. Furthermore, it has the distinction of being a multicentre study conducted in the large city lagoon (Abidjan) and forest (San Pedro) and of providing data on the prevalence and clinical presentation of malaria in PLHIV never previously undertaken in Côte d’Ivoire. This study also took into account the malaria treatment strategy for parasitologically-confirmed cases as recommended by WHO for malaria-endemic settings [18]. The results may therefore differ from those realised in the general population [19-22] because our study was carried out in an HIV population**.**

The profile of patients in our study was similar to that reported by other studies from sub-Saharan Africa [23-25]. Female predominance confirmed the feminisation of HIV infection in this region of Africa according to WHO [26]. The median age of patients was between 30 and 40 years old representing the economic age bracket and this has serious socio-economic impact on the nation. Moreover, only 15% of these patients used an LLIN to protect themselves from mosquito bites, despite LLIN free distribution and awareness campaigns carried out by the National Malaria Control Program among urban and rural communities. Although it is less frequently used overall, LLINs seemed to be more frequently used by San Pedro patients than by Abidjan patients (p<0.001). These findings confirm those of the 2011-2012 national survey, which revealed that the rate of national LLIN use was 17% in Abidjan and 25-47% in the other regions of the country [8]. National malaria and HIV programmes should carry out integrated activities to make LLINs available in HIV treatment centres and strengthen awareness-raising strategies for their use. This because LLINs are known to be an effective means of malaria prophylaxis by virtue of their physical barrier and insecticidal effects [27-29]. This effect was not found in our analysis, perhaps due to the small sample size (Table 2).

Given the fact that 80% of the patients were on cotrimoxazole prophylaxis, which is known to have antiplasmodial activity [6,7], this could explain the low positivity of thick blood films within the study population. In any case, the integrated use of LLINs, cotrimoxazole prophylaxis and antiretroviral therapy is recommended to further reduce the incidence of malaria in the population of PLHIV threatened by high morbi-mortality [10].

Our study revealed that the 10% prevalence of malaria was low and that *P. falciparum* was the only incriminated species. This relatively low prevalence could have been due to daily use of cotrimoxazole prophylaxis that was recommended for all symptomatic and asymptomatic adults and children living with HIV. Cotrimoxazole prophylaxis has been documented to offer protection against malaria among HIV individuals [6,7,30]. In our study, 80% of the patients took cotrimoxazol over a median duration of eight months with extremes of seven days and forty-two months. The low prevalence of malaria has been found by other authors whose studies were similar to ours. Ojurongbe *et al.* [23] found among two hundred HIV-positive patients enrolled that 20 (10%) were positive for malaria by microscopy. Manyanga *et al.* [31] reported a 4.5% prevalence of malaria among 420 HIV-infected pregnant women receiving prophylaxis with cotrimoxazole. Women not adherent to cotrimoxazol prophylaxis were more likely to have malaria infection. However, the risk factors remain severe immunosuppression (OR=3.44), absence of cotrimoxazole prophylaxis (OR=47.45) and absence of antiretroviral tritherapy (OR=3.93) in our study. These findings are consistent with those of Kamya *et al.* [28] and Mermin *et al.* [30] who reported a higher malaria incidence in patients with low CD4 counts and the protective effect of regular cotrimoxazole or even antiretroviral intake against the risk of malaria.

Clinically speaking, uncomplicated malaria was the most observed form (68%). This could be due to early diagnosis and treatment related to early consultations promoted by the awareness/information campaigns that were carried out among the patients of the three study sites six months before starting the study. Our study revealed that early consultation reduces severe forms of malaria in the HIV population. Sensitisation to early consultation should be done in all HIV clinics. The signs of severe malaria were dominated by severe anaemia, which is also the most reported sign in other African studies [22,25]. In addition to haemolysis caused by *Plasmodium*, the mechanism of this anaemia could lead to chronic inflammatory cytokine response due to HIV and/or comorbidities, the agents of opportunistic infections, but also the toxic effect of molecules such as cotrimoxazole and zidovudine (AZT) [32,33].

## 5 Conclusion

This study showed a low prevalence of malaria among febrile HIV-infected patients in HIV clinics. Severe immunosuppression, absence of cotrimoxazole prophylaxis and ART were associated with the occurrence of malaria. Given the low prevalence of malaria in HIV-infected patients, which could be partly due to cotrimoxazole prophylaxis, it is crucial to urge practitioners to confirm diagnosis before initiating malaria treatment as recommended by WHO. The integrated recommendations for cotrimoxazole prophylaxis, LLIN use and early treatment of HIV infection among patients could tremendously reduce malaria morbidity in the population of people living with HIV.
